# Identification of QTLs Controlling Salt Tolerance in Cucumber (*Cucumis sativus* L.) Seedlings

**DOI:** 10.3390/plants10010085

**Published:** 2021-01-03

**Authors:** Dongrang Liu, Shaoyun Dong, Kailiang Bo, Han Miao, Caixia Li, Yanyan Zhang, Shengping Zhang, Xingfang Gu

**Affiliations:** Institute of Vegetables and Flowers, Chinese Academy of Agricultural Sciences, Beijing 100081, China; 82101192231@caas.cn (D.L.); dongshaoyun@caas.cn (S.D.); bokailiang@caas.cn (K.B.); miaohan@caas.cn (H.M.); 82101182236@caas.cn (C.L.); 82101182238@caas.cn (Y.Z.)

**Keywords:** cucumber seedlings, salt stress, quantitative trait loci, candidate genes

## Abstract

Cucumber is very sensitive to salt stress, and excessive salt content in soils seriously affects normal growth and development, posing a serious threat to commercial production. In this study, the recombinant inbred line (RIL) population (from a cross between the salt tolerant line CG104 and salt sensitive line CG37) was used to study the genetic mechanism of salt tolerance in cucumber seedlings. At the same time, the candidate genes within the mapping region were cloned and analyzed. The results showed that salt tolerance in cucumber seedlings is a quantitative trait controlled by multiple genes. In experiments carried out in April and July 2019, *qST6.2* on chromosome six was repeatedly detected. It was delimited into a 1397.1 kb region, and nine genes related to salt tolerance were identified. Among these genes, *Csa6G487740* and *Csa6G489940* showed variations in amino acid sequence between lines CG104 and CG37. Subsequent qRT-PCR showed that the relative expression levels of both genes during salt treatment were significantly different between the two parents. These results provide a basis for the fine mapping of salt tolerant genes and further study of the molecular mechanism of salt tolerance in cucumber seedlings.

## 1. Introduction

Salt-affected land is continually expanding throughout the arable lands of the world, and it is estimated that more than one billion hectares of land are threatened by salt stress, accounting for around 10% of all global land area [1]. Cucumber (*Cucumis sativus* L.) is an annual herbaceous plant in the *Cucurbitaceae* family that originated from the southern foothills of the Himalayas [2]. Cucumber is very sensitive to salt stress, and saline soils significantly reduce the normal growth of cucumber seedlings and the subsequent commercial yield of the crop [3].

The damage caused by salt stress to cucumber seedlings is manifested as an inhibition of plant height and root growth, reduction in water uptake, leaf chlorosis, wilting, and, in extreme cases, death of the seedlings [4]. Studies have shown that concentrations of NaCl higher than 1.2 dS·m^−1^ significantly inhibit the growth of cucumber seedlings at 25 °C [5]. The extent of these effects can be used as an indicator to investigate the salt tolerance of cultivars of cucumber seedlings.

The mining of genes tolerant to salt stress in cucumber seedlings can greatly aid in the understanding and manipulation of growth of cucumber under saline conditions. However, the genetic inheritance of salt tolerance in cucumber is very complicated. Li and Si [6] studied the inheritance of salt tolerance using the highly tolerant inbred line M8 (P1) and the sensitive inbred line M7 (P2). They found that tolerance to 150 mmol·L^−1^ NaCl was controlled by one major gene in addition to polygenes. Wang [7] found that salt tolerance in a F_2_ segregating population conformed to a normal distribution, indicating that cucumber salt tolerance is a quantitative trait controlled by multiple genes, fitting an Additive-Dominance model mainly controlled by gene additive effects. Thus, salt tolerance of cucumber is a complex quantitative trait and the quantitative trait loci (QTL) mapping approach would be an effective way to detect the genes involved.

Salt stress tolerance is an important agronomic trait for many crops, and numerous studies have been reported in *Arabidopsis* and rice, revealing a large number of genes or QTLs that function in response to salt stress, some of which have been cloned. As early as 1999, Gong, et al. [8] used a doubled haploid rice population to identify a major QTL (Std) related to salt tolerance at the seedling stage, which was on chromosome one. After that, more QTLs or genes were identified in *Arabidopsis*, rice, cabbage, and tomato [9,10,11,12,13,14]. However, no QTLs or genes for salt tolerance have been reported in cucumber, and the mechanism for salt tolerance is still not well understood. Therefore, improving the salt tolerance of cucumber seedlings is an important goal of cucumber breeding to reduce yield loss.

The objective of this study was to identify QTLs for salt tolerance in cucumber using a recombinant inbred line (RIL) population constructed from the salt tolerant line CG104 and the salt sensitive line CG37 and analyze candidate genes. The results from this study will promote the breeding of salt tolerant cucumber varieties by marker-assisted selection (MAS).

## 2. Results

### 2.1. Phenotypic Variations and Correlation Analysis

**Figure 1:**
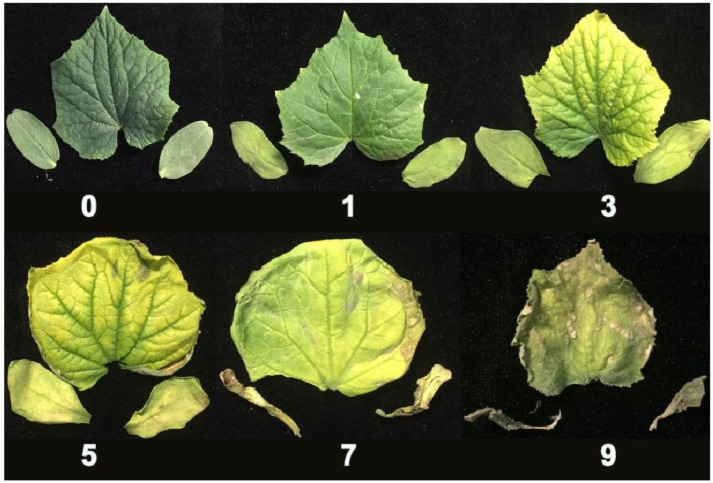
The classification standard to grade salt injury in cucumber seedlings. 0, the cotyledons and the first true leaf show no symptoms of salt injury; 1, the cotyledons are slightly yellowed; 3, the cotyledons and the first true leaf are significantly yellowed; 5, the cotyledons are yellowed and wilted, and the first true leaf is significantly yellowed and wilted; 7, the cotyledons are dehydrated, the large areas of first true leaf are yellowed and wilted; 9, both the cotyledons and the first true leaf are yellowed and dehydrated.

**Figure 2:**
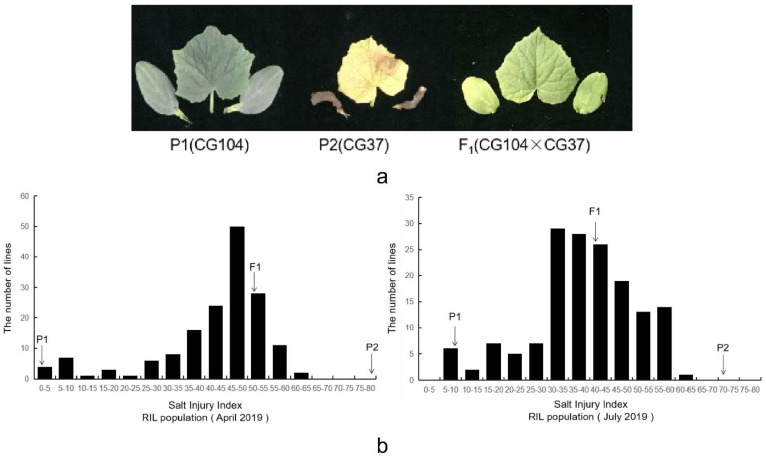
Frequency distribution of salt injury index among the recombinant inbred line (RIL) population. (**a**) Performance of the resistant parental line ‘CG104’, the sensitive line ‘CG37’ and their F_1_ hybrid progeny at 7 d after 200 mmol·L^−1^ NaCl treatment; (**b**) Frequency distribution of the salt injury index among the ‘CG104’ × ‘CG37’ RIL population in two treatments. The abscissa indicates the salt injury index of the RIL population, the ordinate indicates the number of lines. The frequency distributions in April 2019 and July 2019 each presented a continuous distribution from resistant to sensitive phenotypes.

For each of the cucumber seedlings grown under 200 mmol·L^−1^ NaCl, the phenotypic data was collected for the degree of salt injury, and the salt injury index. After the salt treatment, the salt injury was divided into six scales, based on the chlorosis and wilting degree of the first true leaves and cotyledons (Figure 1). In the two experiments, the parental lines, F_1,_ and RIL population all showed different amounts of salt injury. The salt tolerant parental line (CG104), exhibited no symptoms of salt damage seven days after salt treatment, with salt injury indices of 2.3 in April 2019 and 8.0 in July 2019. However, the sensitive parental line (CG37) showed severe leaf chlorosis and wilting, with salt injury indices of 79.5 in April 2019 and 72.0 in July 2019. The F_1_ had salt injury indices of 51.0 in April 2019 and 41.0 in July 2019 (Table 1 and Figure 2). For the RIL population, the salt injury index values showed a continuous and near-normal distribution, suggesting that salt tolerance in cucumber is a quantitative trait (Figure 2).

### 2.2. Linkage Map Construction

**Figure 3:**
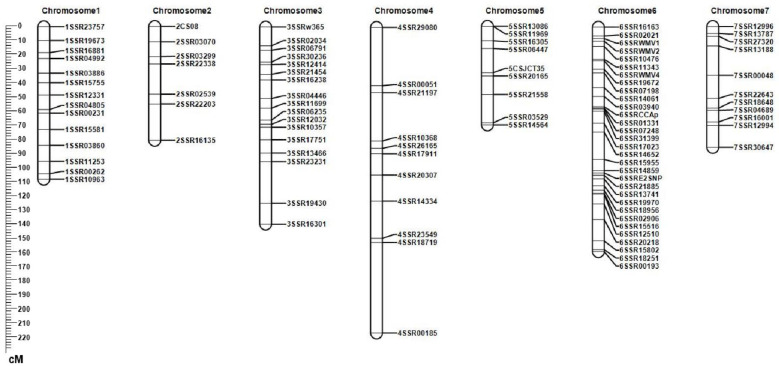
Linkage map of the recombinant inbred line (RIL) population. The tick mark represents the position marked on the chromosome.

Among the 1228 SSR primers used to screen polymorphisms in the parents, 509 (39.52%) were found to be polymorphic. One hundred of these primers were selected to map onto seven linkage groups that covered 863.46 cM. The average distance between the markers was 8.6 cM, and each linkage group was distributed across approximately 7–31 markers (Figure 3). According to SSR marker data and published cucumber map data, the seven linkage groups in the RIL population were assigned to seven cucumber chromosomes according to their physical positions [15,16].

### 2.3. QTL Mapping and Analysis

**Figure 4:**
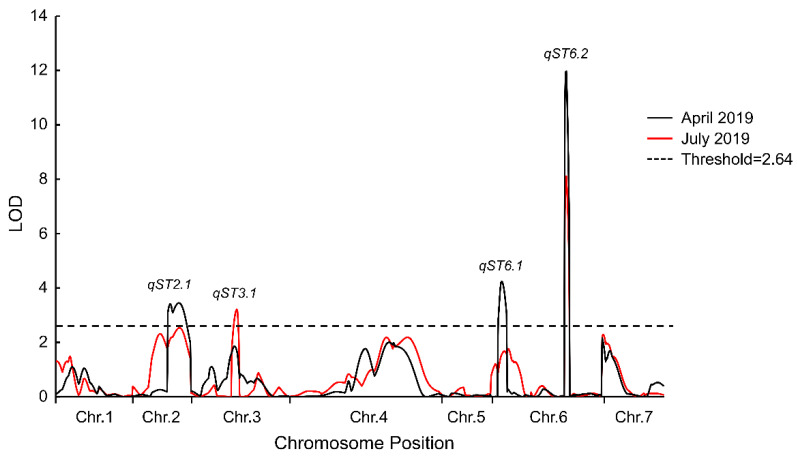
Identified quantitative trait loci (QTL) controlling salt tolerance in cucumber seedlings. The black line indicates the experiment performed in April 2019; the red line indicates the experiment performed in July 2019. *qST6.2* on chromosome six was detected in both April 2019 and July 2019.

The phenotypic data, together with the genetic maps of the RIL population, were used to detect QTLs for salt tolerance (Supplementary Tables S1 and S2). The results showed that when the results for April 2019 and July 2019 were combined and tested with a logarithm of odds (LOD) of 2.64, four QTLs were detected. These were *qST2.1, qST3.1, qST6.1,* and *qST6.2.* Among these loci, *qST6.2* was repeatedly detected in both treatments. The 4.85 cM region was delimited by SSR markers 6SSR13741 and 6SSR19970. The LOD scores in April and July 2019 were 11.97 and 8.11 respectively, also accounting for 21.32% and 18.71% of the phenotypic variation respectively (Table 2 and Figure 4). This region was considered to be the major QTL for salt tolerance in cucumber seedlings.

### 2.4. Prediction and Molecular Cloning of the Candidate Gene

**Figure 5:**
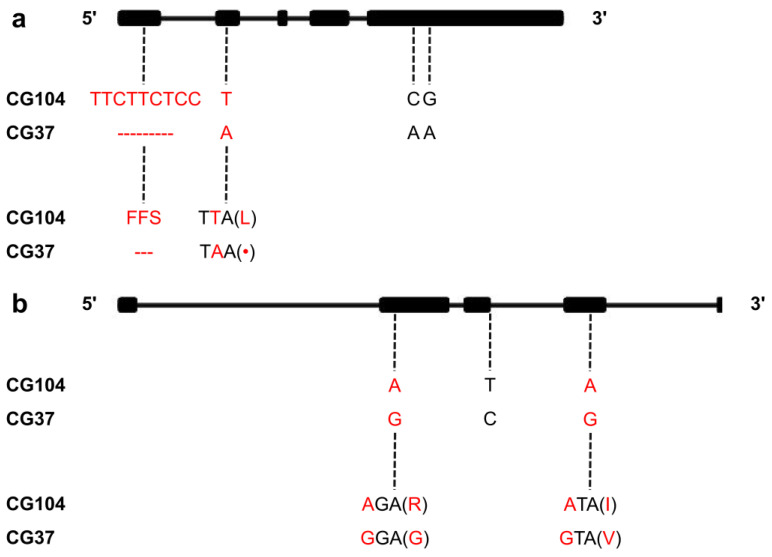
Sequence analysis of candidate genes between CG104 and CG37. (**a**) sequence analysis of *Csa6G487740*. The deletion of nine bases (TTCTTCTCC) on exon one caused a deletion of three amino acids, and the one base substitution (T/A) on exon two caused a nonsense mutation (Leu /·). (**b**) sequence analysis of *Csa6G489940*. The substitution of two bases (A/G, A/G) in exon two and four caused two amino acid substitutions (Arg/Gly, Ile/Val).

Based on the data from the Cucurbit Genomics Database (http://cucurbitgenomics.org/), the mapped QTL was delineated in a region of 1397.1 kb on chromosome six. The functions of 204 genes in the region were predicted by BLAST in the CuGenDB, Swiss-Prot, TAIR, and Gene Ontology (GO) databases (Supplementary Table S3). The functions of nine of these genes were related to salt tolerance (Table 3). These genes were cloned from the parents CG104 and CG37, and SnapGene 4.26 was used for sequence alignment and analysis. The results showed that *Csa6G487740* had one multiple-base deletion and three single-nucleotide polymorphisms (SNPs) within the coding sequences (CDS), and *Csa6G489940* had three SNPs within the CDS (Figure 5). Gene annotation revealed that *Csa6G487740* had five exons, all of which encoded a predicted protein of 569 amino acids. The multiple-base deletion caused the deletion of three amino acids on exon one, and the SNP on exon two resulted in a stop codon. Gene annotation revealed that *Csa6G489940* had five exons, all of which encoded a predicted protein of 294 amino acids. The three SNPs on exon two and four both resulted in changes in amino acids.

### 2.5. Candidate Gene Expression Pattern Analysis

**Figure 6:**
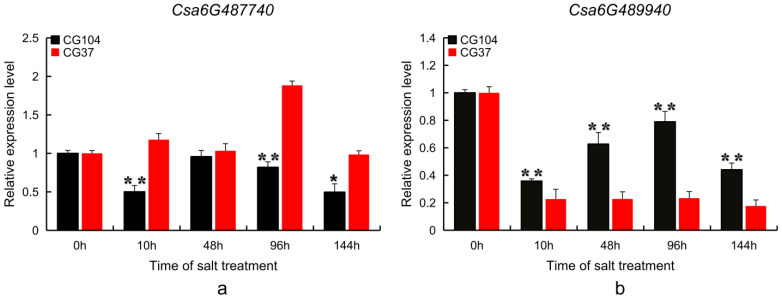
Relative quantitative expression analysis of *Csa6G487740* and *Csa6G489940* in CG104 and CG37. (**a**) Relative quantitative expression analysis of *Csa6G487740*; (**b**) Relative quantitative expression analysis of *Csa6G489940*. Black squares indicate the relative expression levels of the candidate genes at different treatment times (0 h, 10 h, 48 h, 96 h, and 144 h) after salt treatment in CG104; red squares indicate the relative expression levels of candidate genes in CG37; the error bars represent the standard error of three biological replicates; * Significant difference (*p* < 0.05); ** Significant difference (*p* < 0.01).

In order to further identify the candidate genes for salt tolerance, qRT-PCR technology was used to analyze the expression of the two candidate genes using the total RNA extracted at 0 h, 10 h, 48 h, 96 h and 144 h after salt treatment. The relative expression level of *Csa6G489940* was decreased by salt treatment and was significantly different between CG104 and CG37. The relative expression of *Csa6G487740* in CG37 was 2.3 times greater than that in CG104 at 96 h. After salt treatment, and at 96 h, The relative expression of *Csa6G489940* in CG37 showed a decrease of 3.7 times less than that in CG104 (Figure 6). It is proposed that both *Csa6G487740* and *Csa6G489940* are putative candidates in the response to salt stress in cucumber.

## 3. Discussion

The establishment of an efficient system for quantifying salt tolerance in cucumber is essential to obtaining more accurate phenotypic data, which is the basis for QTL mapping. However, there is currently no stable and uniform standard for the identification of salt tolerance in cucumber seedlings. Zhang, et al. [17] took the degree of leaf wilting as the main trait and divided the salt damage into six grades: 0, 1, 2, 3, 4, and 5. Zhang, et al. [18] used leaf epidermal cell mortality as an indicator and proposed the local standard DB13/T 1471–2011 for the identification of cucumber salt tolerance in Hebei Province, but it has not been widely used. In this study, the degree of chlorosis and wilting were combined and divided into six grades: 0, 1, 3, 5, 7, and 9, to establish a new method to identify salt tolerance in cucumber. This method was accurate enough to allow for the identification of the QTLs controlling salt tolerance in cucumber seedlings.

Studies have shown that plant salt tolerance is a quantitative trait, which is controlled by multiple genes and is easily affected by environmental factors [19]. In this study, the RIL population generated from cucumber inbred lines CG104 (salt tolerant) and CG37 (salt sensitive) were used to determine the genetic basis of salt tolerance. The salt injury indices of this segregating population were continuously distributed, which was suitable for establishing a quantitative genetic model. This result indicated that the salt tolerance of cucumber seedlings was controlled by multiple genes, which was consistent with previous studies on cucumber salt tolerance [6,7].

In recent years, many QTLs related to the salt tolerance of major field crops such as rice, soybean, wheat, and maize, as well as vegetable crops such as cabbage and tomato have been reported [13,14,20,21,22,23]. But currently there are few reports on the mining of cucumber salt tolerant genes, and no QTL mapping studies have been carried out. In this study, one QTL on chromosome six was repeatedly detected in the RIL population, defined by markers 6SSR13741 and 6SSR19970 (1397.1 kb).

At present, many QTL loci related to the salt tolerance of crops have been reported, and some major loci have been successfully cloned, such as the *SKC1* gene in rice, and the *CDF1* gene and *SALT3* gene in soybean [24,25,26]. In this study, nine salt tolerance related genes were identified within the major QTL, among which the genes *Csa6G487740* and *Csa6G489940* were found to produce differences in the sequences of amino acids between CG104 and CG37. *Csa6G487740* encodes a mitochondrial transcription termination factor family protein that is homologous to the gene product of *MDA1* (*MTERF DEFECTIVE IN Arabidopsis 1*), which plays a role in chloroplast development and abiotic stress response such as salt stress in *Arabidopsis* [27,28,29]. *Csa6G489940* encodes a B3 domain-containing protein, and a protein in this family was detected to be expressed in *Arabidopsis thaliana* after salt stress treatment [30]. In this study, the expression level of both *Csa6G487740* and *Csa6G489940* were decreased by salt treatment and there were significantly differences between the salt tolerant parent CG104 and the salt sensitive parent CG37. Both of these genes appear to be involved in the response to stress imposed by NaCl on cucumber. The candidate genes identified will be further studied to understand the molecular mechanism underlying salt tolerance in cucumber, which will effectively lead to the breeding of salt resistant cultivars in the future.

## 4. Materials and Methods

### 4.1. Plant Materials and Population Development

Plant material was developed from a hybrid of CG104 (a South China type that is salt tolerant) and CG37 (a salt sensitive type from Russia). CG104 and CG37 were crossed to obtain F_1_ plants, F_1_ were self-pollinated to produce F_2_ offspring, and 165 F_8_ RILs were then generated from the resultant F_2_ plants by the single-seed propagation method under natural field conditions at the Institute of Vegetables and Flowers, Chinese Academy of Agricultural Science at Beijing (39°9′ N, 116°3′ E).

### 4.2. Evaluation of Salt Tolerance Ability and Data Analysis

Salt treatment experiments were carried out in April 2019 and July 2019 in the same plastic greenhouse at the Institute of Vegetables and Flowers, Chinese Academy of Agricultural Science at Beijing (39°9′ N, 116°3′ E). Three replicates were set for each experiment, each of which had eight plants. Cucumbers were cultivated in 54 cm × 28 cm × 6 cm soil pots, with trays for water collection at the bottom. There was sufficient nursery substrate in the soil pots. At the stage of one-mature-leaf, the seedlings were exposed regularly to two liters of 200 mmol·L^−1^ NaCl to ensure that each seedling was saturated with the same amount of salt solution. After 7 days of salt treatment, the first true leaves and cotyledons displayed different degrees of chlorosis and wilting. These symptoms within the RIL population were divided into six scales, and the index used was as follows: 0, the cotyledons and the first true leaf show no symptoms of salt injury; 1, the cotyledons are slightly yellowed; 3, the cotyledons and the first true leaf are significantly yellowed; 5, the cotyledons are yellowed and wilted, and the first true leaf is significantly yellowed and wilted; 7, the cotyledons are dehydrated, the large areas of first true leaf are yellowed and wilted; 9, both the cotyledons and the first true leaf are yellowed and dehydrated. The salt injury index was calculated as below: salt injury index = S × ∑(s × n)/N. Where “S” represents the highest salt injury rating scale, “s” represents the salt injury grade, “n” represents the number of plants in the salt injury grade, and “N” represents the total number of plants.

### 4.3. Molecular Marker Analysis

Total DNA of the 165 RILs and the two parental lines was extracted from the first fresh leaf of each one-mature-leaf stage seedling using the CTAB method of Doyle and Doyle [31]. The DNA concentration and quality were examined by electrophoresis on a 1% (*w/v*) agarose gel and NanoDrop One (Thermo Scientific, Waltham, MA, USA). A total of 1228 SSR primers from Shi, et al. [16] were used to screen for polymorphisms between the two parental lines. The Polymerase Chain Reaction (PCR) amplification and polyacrylamide gel electrophoresis (PAGE) refer to Song [2]: the 10 μL mixture system of PCR contains 3 μL of total DNA (15 ng·μL^−1^), 1 μL each of forward and reverse primers (10 μmol·L^−1^), and 5 μL of 3G Taq Master Mix for PAGE (Vazyme, Nanjing, China); the PCR reaction program in the S1000 Thermal Cycler (Bio-Rad, Hercules, CA, USA) is 95 °C for 3 min, 32 cycles (95 °C for 15 s, 55 °C for 15 s, and 72 °C for 30 s), and a final extension at 72 °C for 5 min; amplified products are separated by 6% PAGE at 150 V for 1 h, and the bands are visualized and photographed after silver staining. Amplified DNA fragments with polymorphisms were used for the analysis of the RILs and linkage mapping.

### 4.4. Linkage Map Construction and QTL Analysis

Polymorphisms were entered into WPS Office (Kingsoft, Beijing, China), where the band pattern of the maternal parent (CG104) was marked with “a”, the paternal parent (CG37) was marked with “b”, the heterozygous band was marked with “h”, and missing values were marked with “u” (Supplementary Materials Table S2). The linkage map was constructed using QTL IciMapping 4.1 (CAAS, Beijing, China) [32,33], and the minimum logarithm of odds (LOD) was set as 3.0. The genetic distance between markers was calculated using the Kosambi map function [34], and the linkage groups were assigned to chromosomes 1–7 according to the published markers [35,36]. Inclusive composite interval mapping (ICIM) analysis was performed to identify QTLs and the LOD threshold was determined by computing 1000 permutations at *p* = 0.05 level, as implemented by the QTL IciMapping 4.1 [32,33]. Each chromosome was scanned for the possibility of QTL at intervals of 1 cM. The name of the locus consists of three parts: the abbreviation of the trait, the chromosome (Chr.) number, and the locus number [37].

### 4.5. Prediction and Molecular Cloning of the Candidate Gene

Based on the sequencing information of the cucumber genome database website (http://cucurbitgenomics.org/), the physical location of the locus was determined, and the genes within the confidence interval were identified. Gene function was analyzed and predicted by BLAST in the CuGenDB, Swiss-Prot, TAIR and Gene Ontology (GO) databases to identify the candidate genes [16]. The candidate genes were selected based on the gene annotation and the function of its homolog in other plants. Primer Premier 6.0 software (Premier Biosoft, San Francisco, CA, USA) was used to design primers (Supplementary Materials Table S3). PCR amplification was performed with 2 × Phanta Max Master Mix (Vazyme, Nanjing, China), and the PCR products were sequenced by Sangon Biotech. The candidate genes in the parents CG104 and CG37 were amplified by PCR, and SnapGene 4.26 was used for sequence alignment and analysis.

### 4.6. Extraction of Nucleic Acids and qRT-PCR

Using the parents CG104 and CG37 as experimental materials, a total of five time points were set, namely 0 h, 10 h, 48 h, 96 h, and 144 h after salt treatment. The first true leaf of each seedling was harvested, and immediately put into liquid nitrogen, and stored at −80 °C. The leaves of three seedlings were mixed together as a sample, and each material had three samples at each time point. Taking 100 mg of leaves from each sample that were ground into powder in liquid nitrogen for RNA extraction, the TaKaRa MiniBEST Universal RNA Extraction Kit (TakaRa, Kusatsu, Japan) was used to extract the total RNA. The RNA concentration and quality were examined by electrophoresis on a 1% (*w/v*) agarose gel and NanoDrop One (Thermo Scientific, Waltham, MA, USA). Reverse transcription of the extracted RNA into cDNA was performed with the UEIris II RT-PCR System (Biodee, Beijing, China). The qRT-PCR with *Actin* (*CsaV3_6G041900*) and *Ubiquitin* (*CsaV3_5G031430*) as reference genes were performed independently, and their results were normalized using the geometric mean. According to the CDS sequence on the reference genome of the candidate gene and two reference genes, primer 6.0 software was used to design primers (Supplementary Table S3). The 20 μL mixture system of qRT-PCR contained 2 μL of cDNA (50 ng·μL^−1^), 0.4 μL each of forward and reverse primers (10 μmol·L^−1^), 7.2 μL ddH_2_O, and 10 μL of 2 × ChamQ Universal SYBR qPCR Master Mix (Vazyme, Nanjing, China). The PCR reaction program in the CFX96 Real-Time System (Bio-Rad, Hercules, CA, USA) is 95 °C for 30 s, 40 cycles (95 °C for 10 s, 60 °C for 30 s), melt curve 60 °C to 95 °C, and increment 0.5 °C. Analysis of the relative expression data of the candidate genes was performed using the 2^−ΔΔCt^ method [16].

## 5. Conclusions

We reported that the salt tolerance in cucumber seedlings is a quantitative trait controlled by multiple genes. One locus, namely *qST6.2,* that regulates salt tolerance in cucumber seedlings, was repeatedly detected. Furthermore, candidate genes within *qST6.2* that are involved in the salt stress response were predicted through gene analysis, cloning, and qRT-PCR. This study provides a basis for the fine mapping of salt tolerant genes and further study of the molecular mechanism of salt tolerance in cucumber seedlings.

## Figures and Tables

**Table 1:** The salt injury indexes of the parental lines, F_1_ plants, and recombinant inbred line (RIL) populations.

Time	Parental Lines (Mean ± S.E)		F_1_ Plants	RIL Populations			
	‘CG104’	‘CG37’	(Mean ± S.E)	Mean ± S.E	SD ^1^	Skewness	Kurtosis
April 2019	2.25 ± 0.12	79.50 ± 0.24	51.00 ± 0.31	42.03 ± 1.03	13.06	−1.54	2.00
July 2019	8.00 ± 0.16	72.00 ± 0.83	41.00 ± 0.56	38.48 ± 0.98	12.26	−0.56	0.21

^1^ SD: standard deviation.

**Table 2:** **Quantitative trait loci** (QTL) controlling salt tolerance and their effect on cucumber seedlings.

Time	QTL	Chr.	Position (cM)	Marker Interval	LOD	R^2^ (%)
April 2019	*qST2.1*	2	66.0	2SSR22203-2SSR16135	3.45	8.35
	*qST6.1*	6	15.0	6SSR10476-6SSR11343	4.24	7.18
	*qST6.2*	6	107.0	6SSR13741-6SSR19970	11.97	21.32
July 2019	*qST3.* *1*	3	65.0	3SSR11699-3SSR06235	3.21	7.20
	*qST6.2*	6	107.0	6SSR13741-6SSR19970	8.11	18.71

**Table 3:** Candidate genes responsible for cucumber salt tolerance with major quantitative trait loci (QTL) *qST6.2*.

Gene ID	Position on Chr.6	Homologous Gene in *Arabidopsis*	Predicated Gene Function
*Csa6G483260*	22147783-22153709	*AT4G33470*	Histone deacetylase, putative
*Csa6G483280*	22159504-22162733	*AT5G63980*	3’(2’),5’-bisphosphate nucleotidase like protein
*Csa6G486760*	22594296-22600877	*AT2G17520*	Serine threonine protein kinase, putative
*Csa6G487000*	22792092-22793544	*AT1G16850*	Transmembrane protein
*Csa6G487690*	22933332-22934682	*AT2G23780*	RING finger protein
*Csa6G487740*	22955329-22957793	*AT4G14605*	Mitochondrial transcription termination factor family protein
*Csa6G488350*	23038795-23040540	*AT3G50830*	Cold acclimation protein-like protein
*Csa6G489940*	23128924-23132268	*AT4G34400*	B3 domain-containing protein
*Csa6G490230*	23301401-23303177	*AT1G49570*	Peroxidase

## Data Availability

All relevant data are within this article and its Supplementary Data file.

## Supplementary Materials

The following are available online at https://www.mdpi.com/2223-7747/10/1/85/s1, Table S1: Phenotypic data of the RIL population, Table S2: Genotype data of the RIL population, Table S3: Candidate genes within the major QTL *qST6.2*, Table S4: Oligos used for genes cloning and the qRT-PCR.
